# Enzymatic hydrolysis is limited by biomass–water interactions at high-solids: improved performance through substrate modifications

**DOI:** 10.1186/s13068-018-1339-x

**Published:** 2019-01-04

**Authors:** Noah D. Weiss, Claus Felby, Lisbeth G. Thygesen

**Affiliations:** Department of Geosciences and Natural Resource Management, University of Copenhagen, Frederiksberg, Denmark

**Keywords:** High-solids effect, Enzymatic hydrolysis, T_2_ NMR, Diffusion, Lignocellulose, Pretreatment

## Abstract

**Background:**

To improve process economics for production of fuels and chemicals from lignocellulosic biomass, high solids concentrations are applied in enzymatic hydrolysis, to increase product concentration and reduce energy input. However, increasing solids concentrations decrease cellulose conversion yields, the so called ‘high-solids effect.’ Previous work suggests that product inhibition and mixing contribute, but an understanding of how biomass properties influence the high-solids effect, is lacking.

**Results:**

Cellulose hydrolysis yields with an industrial cellulase (Ctec2) were measured on pretreated wheat straw and spruce from 5 to 30% dry matter (DM), and compared to yields of an older industrial cellulase mixture (Celluclast 1.5L/Novozym188). For Ctec2, yield was independent of DM below 15–18% DM, while yields decreased with increasing DM above this range, but at different rates for each biomass. For Celluclast 1.5L/Novozym188, yields decreased already from the lowest DM, suggesting that the high-solids effect was more a function of product inhibition, while the yields of the newer Ctec2 mixture were driven more by biomass–water interactions. LF-NMR relaxometry showed that the onset of the high-solids effect for Ctec2 corresponded to the disappearance of free water from the system, and a decrease in water self-diffusion rates. While the spruce had higher yields at low-solids, the wheat straw had higher yields at high-solids conditions, exhibiting that relative yields at low and high-solids are not related. Higher yields corresponded to increased water constraint by the biomass at high-solids conditions. Modifications to the pretreated wheat straw resulted in improved yields, and changes to the inflection point and intensity of the high-solids effect, showing that this effect can be reduced.

**Conclusions:**

The high-solids effect is both enzyme and substrate dependent, and can be reduced by modifying the pretreated biomass, suggesting that pretreatment processes can be designed to achieve similar effects. Yields at low and high-solids concentrations do not correlate for a given biomass, and thus industrial evaluation of biomass recalcitrance should be carried out at high-solids conditions.

**Electronic supplementary material:**

The online version of this article (10.1186/s13068-018-1339-x) contains supplementary material, which is available to authorized users.

## Background

As climate change is increasing [1], and the environmental and societal consequences of continued fossil fuel extraction and use are being widely recognized, sustainable and renewable sources of energy, fuels, and chemicals must be developed to replace fossil fuels. A promising near term process for producing liquid fuels and chemicals is by the utilization of lignocellulosic biomass from sustainably managed sources. Through biochemical conversion processes, lignocellulosic biomass can be deconstructed into its constituent carbohydrate components, which can be further fermented by microorganisms to produce fuels and chemicals. However, plants have adapted over millions of years to resist this deconstruction process carried out by fungi or bacteria, a property known as recalcitrance. Existing industrial processes for the deconstruction of lignocellulosic biomass require the input of enzymes, materials and energy, making cost competitive fuels and chemicals difficult to achieve [2].

One method to improve process economics is to increase the amount of biomass solids in the reaction solution during processing, both for the pretreatment, enzymatic hydrolysis and fermentation steps [3]. This is commonly referred to as a ‘high-solids’ process [4], with insoluble solids concentrations above 20% dry matter (DM) [5]. Increasing solid concentrations reduces tank volumes, increases product concentrations, and thereby decreases separation costs and improves both capital and operating costs. While pretreatment at high-solids loadings is fairly straightforward [6, 7], and commercial yeasts are capable of fermentation at high sugar and ethanol concentrations [8], efficient enzymatic hydrolysis remains a challenge at high-solids concentrations. As solids concentrations increase, conversions yields decrease in an apparently linear fashion i.e., the high-solids effect [9, 10]. This effect is well documented [11, 12], and is thought to be caused by a number of factors, from increasing enzyme product and pretreatment-produced inhibitor concentrations [13, 14], to less efficient mixing [15, 16], and mass transfer limitations [17, 18], as well as water availability and water constraint [19, 20]. While many factors are at play in causing the high-solids effect, their relative contributions have yet to be understood. Thus, there remains a need for a better understanding of why the high-solids effect occurs, and how this effect may be ameliorated through changes to the biochemical conversion process.

Some complimentary approaches to overcome the high-solids effect have been previously suggested. First, enzymes selected to deconstruct lignocellulose can be improved to reduce product inhibition and biomass recalcitrance. Significant work has been carried out to this extent. The beta-glucosidase activity has been increased and lytic polysaccharide monooxygenases have been added to commercial cellulase preparations, improving hydrolysis yields also at high-solids concentrations, and reducing product inhibition [21–24]. However, even these advanced enzyme mixtures show decreased yields with increasing solids concentrations [23]. Secondly, improvements can be made in biomass processing with regards to mixing and mass transfer equipment, as seen with the transition to horizontal and free fall mixing hydrolysis reactors, as well as fed batch, continuous hydrolysis, and alternative processes configurations [9, 11, 12, 16]. These process methods have only been demonstrated at 15–20% insoluble solids, and hydrolysis yields were still negatively impacted by increasing solids concentrations. Similarly, simultaneous saccharification and fermentation (SSF) processes can reduce the effects of product inhibition, but at the expense of reducing the process temperature below the optimum for the cellulytic enzymes. Thirdly, and what will be the topic of this paper, is the option to improve the properties of the pretreated biomass itself, such that the high-solids effect is reduced. We hypothesize that physical and chemical properties of the biomass, and specifically the interaction of the biomass with water, contribute to the high-solids effect, and that by understanding and modifying these properties, it will be possible to improve enzyme performance at high-solids loadings.

The interrelation between water and biomass may be a key determining factor for the high-solids effect. At 25–40% DM, there is a stoichiometric excess of water necessary for hydrolysis reactions, but some research has shown increased water constraint and reduced diffusion rates on model substrates [17], and that changing the cellulose conformation changes hydrolysis yields and water constraint at high-solids [19].

Thus, while there is a theoretical background for cellulose water interactions having a significant effect on high-solids hydrolysis yields, this has not been translated into real pretreated lignocellulosic materials, or how changes to the lignocellulosic materials may change these interactions. Previous studies have looked at improving pretreatments to obtain high cellulose and hemicellulose conversion yields [25–27], the majority of these studies evaluate the efficacy of the pretreatment methods at low-solids conditions (below 20% DM), and therefore provide no indication if these pretreatments lead to reduced recalcitrance and improved enzyme performance at high-solids conditions.

In this work, we show the high-solids effect for washed steam pretreated wheat straw and SO_2_ pretreated spruce using commercial cellulase preparations and free fall mixing technology. We observe how cellulose hydrolysis yields decrease with increasing solids, and relate this to biomass–water interactions. Pretreated wheat straw is then physically and/or chemically modified and the effect on hydrolysis yields and biomass–water interactions are observed. To quantify biomass–water interactions and mass transfer, time domain Low Field Nuclear Magnetic Resonance (LFNMR) relaxometry methods are used to characterize water constraint by means of spin–spin (T_2_) relaxation times, and the self-diffusion coefficient of water within the biomass matrix. The LFNMR relaxometry methods [28, 29] have been used extensively to measure water constraint by cellulose [19, 30], wood [31–35], and pretreated biomass [36, 37]. By comparing changes in the biomass–water interactions to changes in the biomass chemistry and structure, and how these may correspond to the high-solids effect, we aim to better understand how pretreatment methods can be tailored to high-solids conditions.

## Results

### Impact of increased DM on hydrolysis yields from two different feedstocks

**Fig. 1:**
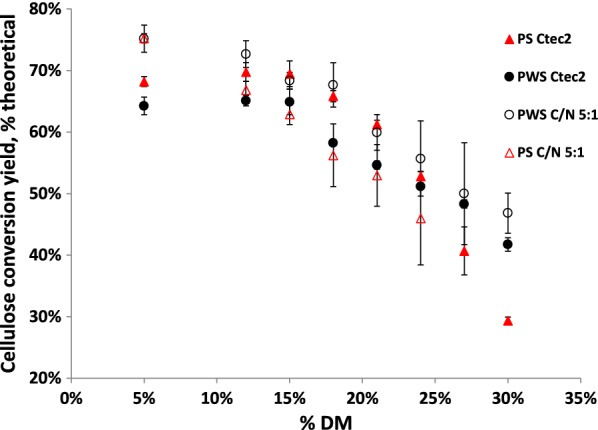
Cellulose conversion yields of pretreated spruce (PS -triangles) and pretreated wheat straw (PWS—circles) materials after enzymatic hydrolysis for 72 h with either 15 mg EP/g cellulose Ctec2 enzyme preparation (filled shapes) or 45 mg EP/g cellulose Celluclast/Novozym188 (C/N 5:1) enzyme mix (hollow shapes), from low to high  % DM. For Ctec2-treated samples, cellulose conversion yields remain stable with DM until 15–18% DM, where they begin to decrease, but at different rates for the two materials. For the C/N-treated samples, reductions in cellulose conversion yields begin even at low DM concentrations. This suggests that high-solids effect is influenced by both the enzyme preparation, and the substrate type. Error bars represent ± 1 standard deviation of triplicate experiments

As a baseline for the high-solids effect, two commercially relevant pretreated washed materials were hydrolyzed over a range of solids concentrations between 5 and 30% DM. Either Ctec2 or Celluclast1.5L/Novozym188 (C/N) enzyme mixtures were applied, and the high-solids effect for the different mixtures was compared. As expected, cellulose hydrolysis yields decreased with increasing DM concentrations for both the pretreated wheat straw (PWS) and pretreated spruce (PS) materials as well as for both enzyme preparations (Fig. 1). For the samples hydrolyzed with the C/N enzyme mixture, a decrease in hydrolysis yields was observed already from low-solids concentrations, albeit at different rates of decrease for the two pretreated feedstocks. For the samples hydrolyzed with Cetec2, the decrease in yield was not immediately observed with increasing solids concentrations, but first occurred at 15–18% DM for both materials, with hydrolysis yields being relatively constant at lower solids concentrations. At solids concentrations above the observed inflection point, hydrolysis yields decreased for both materials, however, at different rates of decrease, with conversions yields of 42% and 29% for PWS and PS, respectively. This difference in rate of decrease led to an inversion in relative yields for the two materials, with PS having higher hydrolysis yields at lower solids concentrations, and PWS having higher yields at higher solids concentrations for the Ctec2-treated samples. These results point to the high-solids effect being dependent on both the enzyme mixture used, and the properties of the pretreated materials. The C/N enzyme mixture, a cocktail used in older studies of the high-solids effect [9, 10], exhibited the high-solids effect already from low DM concentrations, while the Ctec2-treated samples exhibited the high-solids effect first at higher DM concentrations. This suggests that there are different factors governing the high-solids effect for the two enzyme preparations. Similarly, the difference in the onset and intensity of the high-solids effect for the two different feedstocks raises the question of what factors lead to the observed relationship between DM and hydrolysis performance, and specifically what biomass characteristics and biomass–water interactions might lead to improved yields at higher DM concentrations.

### Water constraint profiles

**Fig. 2:**
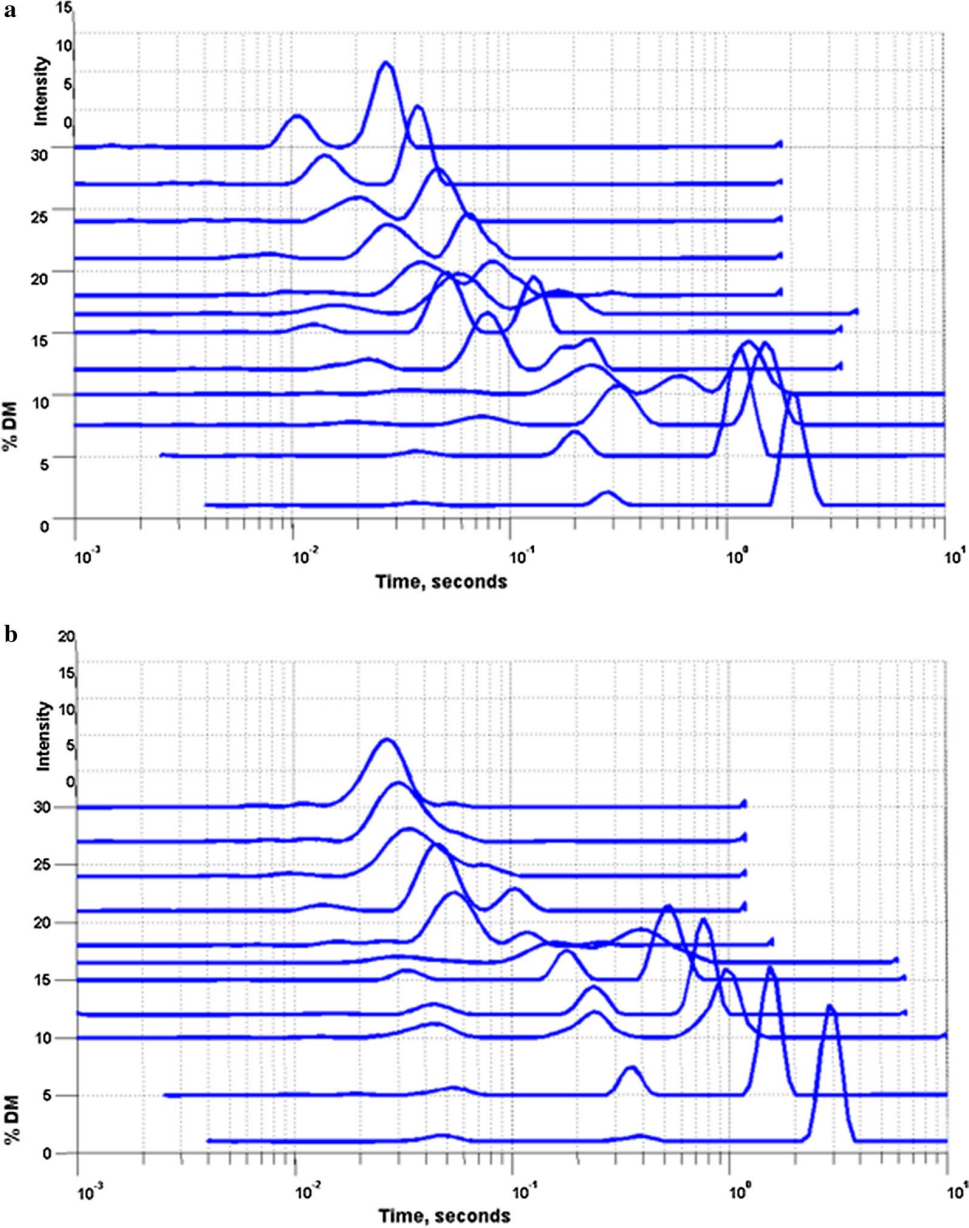
LFNMR T_2_ profiles for PWS (**a**) and PS (**b**) pretreated materials at increasing solids concentrations (% DM), after analysis with NNLS to assess the T_2_ times and relative amounts of water at different relaxation rates. X axis is log scale, and peak height is expressed as intensity, with arbitrary units. Triplicate samples were measured, and their average is given

To determine how biomass–water interaction change with increasing solids, LFNMR was applied to measure the relaxation time of the water hydrogen protons. The T_2_ relaxation times obtained by fitting each relaxation curve by a single exponential decay decreased with increasing solids concentrations, though to different degrees for the two pretreated materials, with PWS having shorter relaxation times at a given  % DM (Additional file 1: Table S1). However, the single component T_2_ times only give one value for the entire system, and not a detailed picture of biomass–water interactions in the system. A non-negative least squares (NNLS) regression algorithm was used to separate the relaxation times of the different water populations present in the system. NNLS analysis of the T_2_ decay curves of the PWS- and PS-pretreated feedstocks at different DM showed multiple distinct pools of constrained water, and both the size, relative amounts of water, and the individual T_2_ relaxation times of these pools changed with increasing solids concentrations (Fig. 2). Below 10% DM, both samples showed large peaks with long relaxation times (≥ 1000 ms), accounting for the majority of the water in the sample (Additional file 1: Table S1). This peak was identified as free water and is assumed to be minimally constrained by the biomass. This large free water peak disappeared with increasing solids concentrations, above 12% and 16.5% DM for PWS and PS, respectively. Interestingly, this corresponded well to the solids concentrations where an initial decrease in hydrolysis yields is seen for the respective biomasses hydrolyzed with Ctec2. Thus, the disappearance of free water from the hydrolysis reaction system correlates to the initiation of the high-solids effect.

Above the solids concentration where free water with long relaxation times could no longer be detected, PS showed one major pool of water only, for which the T_2_ relaxation time decreased with increasing solids concentrations, having a relaxation time of 26 ms at 30% DM. PWS showed a similar decrease in T_2_ relaxation times, but the majority of the water was divided into two separate pools, with T_2_ times of 26 and 11 ms at 30% DM. Furthermore, the rate of decrease in T_2_ relaxation times was greater for PWS than for PS, suggesting that water was more constrained and thus in closer contact with the PWS at increasing solids concentrations. When comparing these data to the corresponding hydrolysis yields, it can be seen that PWS with its more constrained water had higher hydrolysis yields than the PS. Thus, increased water constraint by the biomass, especially at high-solids concentrations, correlates to higher hydrolysis yields.

### Diffusivity of water with increasing solids concentrations

**Fig. 3:**
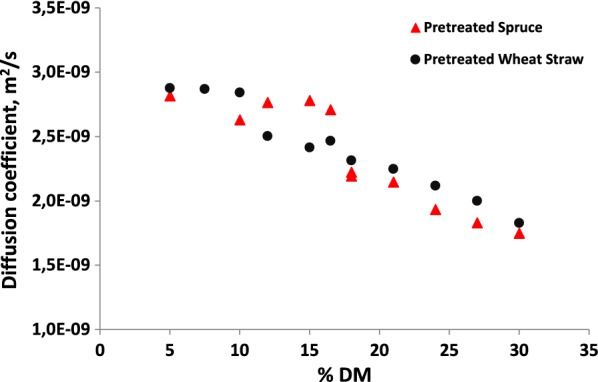
Diffusion coefficients for water at increasing solids concentrations (% DM) of PWS and PS samples, as measured by LF-NMR. Data points represent average measurements for 3 individual samples. Standard deviations are below the size of the markers

The diffusivity, as represented by the self-diffusion coefficient of water has been shown to decrease with increasing solids concentration [17], and has long been thought to impact biomass hydrolysis yields at high-solids [10]. Figure 3 shows internal diffusion rates for water for PS and PWS at increasing solids concentrations. Similar to the hydrolysis yield data, a plateau in diffusion rates at lower solid concentrations was seen for both the PS and PWS samples, with diffusion rates first decreasing at the same % DM as the decrease in hydrolysis yields (Fig. 1) and a lower level of free water (Fig. 2) were seen. The relationship between the lack of free water and diffusion is straightforward considering that the diffusion measurement is an average for all water in the system, and where free water dominates, the average diffusion rate will not significantly change until the free water pool is depleted. After the inflection point, diffusion rates decreased, with PWS having a slightly higher diffusion rate than PS, again correlating to hydrolysis yields.

### Effects of substrate modification

To investigate how chemical or physical modifications to the substrate may correlate to biomass–water interactions, modifications were done by three different treatments of the PWS substrate; a simple size reduction by knife milling of the wet material (PWS milled), a delignification of the PWS material (PWS de-lignified), and incubation with a xylanase enzyme mixture to remove some hemicellulose from the biomass matrix (PWS xylanase). The treatments employed to produce these changes in the biomass are not suggested as specific industrial treatments for biomass after pretreatment, but were used to induce distinct changes to the biomass. Compositional changes to the de-lignified PWS- and xylanase-treated PWS are given in Table 1.

**Table 1:** Extractive free and washed composition for pretreated and modified biomasses

Biomass	Glucan (%)	Xylan (%)	Mannan	Lignin (%)	Ash (%)
PS	48	0.3	0.4%	50	0.1
PWS	54	4	0	34	6
De-lignified PWS	86	6	0	10	5
Xylanase PWS	55	3	0	41	6

**Fig. 4:**
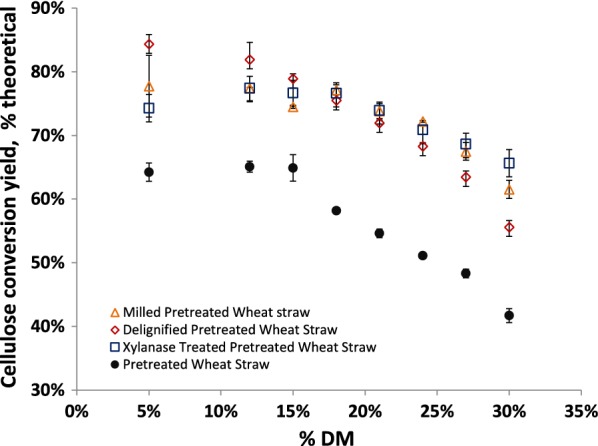
Cellulose to glucose conversion yields for modified PWS samples (milled PWS [triangle], de-lignified PWS [diamond], xylanase-treated PWS [square], and unmodified PWS [filled circle]) hydrolyzed for 72 h with Ctec2 at different  % DM. All modified materials showed improvement over the untreated PWS, however they responded differently to increasing % DM. Data points are averages of triplicate experiments, and error bars represent the standard deviation between experiments

Enzymatic hydrolysis experiments for the modified materials were done under the same conditions as for the non-modified PWS material, and cellulose to glucose conversion yields measured (Fig. 4). All of the modified samples had higher cellulose conversion yields than for the unmodified PWS. A general high solids effect was observed for the modified samples, with cellulose conversion yields initially remaining constant with increasing solids concentrations, and then decreasing above a given solids concentration. However, the % DM above which yields began to decrease was different for the modified samples, as well as the rate of decrease in cellulose conversion yields with increasing % DM. The milled PWS had a similar rate of decrease in yield with increasing solids concentrations as the unmodified PWS; however, the inflection point for the milled PWS was at a higher solids concentration (18% DM) than for the unmodified PWS (15% DM). The xylanase-treated PWS had a slower rate of decrease in cellulose conversion yields as compared to the unmodified material, with the inflection point also located at 18% DM. Interestingly, the de-lignified PWS, which had the highest yields of all the materials at low-solids concentrations, had an inflection point at 12% DM. The inflection point may have occurred at lower solids concentrations, but no experiment was run between 5 and 12% DM. For the de-lignified PWS, cellulose conversion yields decreased more quickly than for the unmodified PWS. Notably, the xylanase-treated PWS had the lowest cellulose conversion yields of the modified samples at low-solids concentrations, but had the highest yields at 30% DM. Thus, also this experiment showed that the hydrolysis performance of a given material at low-solids concentrations cannot be compared to its performance at high-solids concentrations. The implication is that it is important to evaluate pretreated materials at a relevant solids concentration for the enzymatic process in question. The experiment also showed that simple chemical and physical changes to the biomass can lead to a modified, and in some cases, a reduced high-solids effect.

### Effect of PWS modifications on LFNMR relaxometry water constraint profiles

**Fig. 5:**
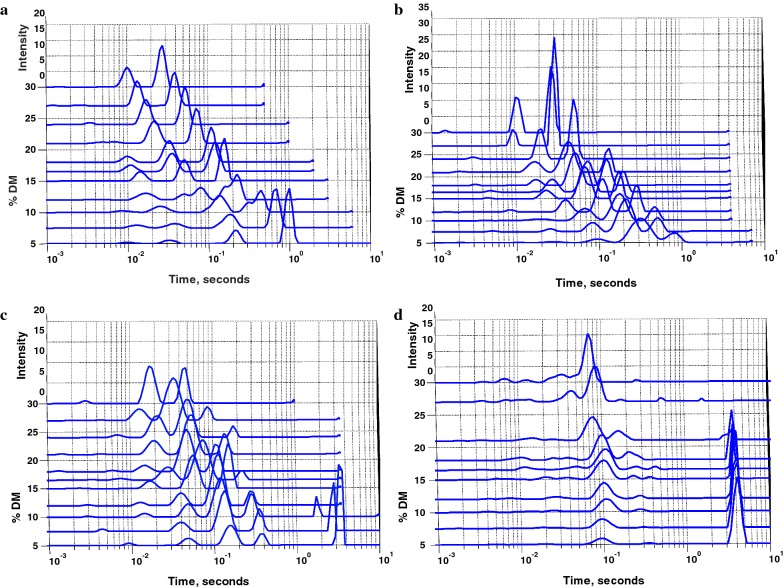
LFNMR T_2_ profiles for **a** milled PWS, **b** de-lignified PWS, **c** xylanase-treated PWS, and **d** Avicel, at different solids concentrations (% DM), after NNLS analysis, with each peak representing a pool of water at a given constraint environment, and the area of the peak the relative amount of water in that pool within a given sample. Spectra are representative of triplicate samples

T_2_ NMR profiles of the modified materials at increasing % DM showed similar general trends as the unmodified PWS, however with some distinct differences (Fig. 5). The milled PWS sample T_2_ profile was very similar to the unmodified PWS, in that there were two major peaks identified with the biomass. However, the free water peak in general had shorter relaxation times and appeared to merge with the next shortest peak above 15% DM and had a significant decrease in relaxation time with increasing solid concentrations below 15% DM (Fig. 5a). Above 15% DM, the relaxation times of the two major peaks decreased significantly as with the unmodified PWS, having identical T_2_ times at 30% DM (10 ms and 27 ms), but with relatively more water in the more constrained peak for the milled PWS than for the unmodified PWS (Additional file 1: Table S1).

The de-lignified PWS sample showed significant changes to the T_2_ profiles as compared to the unmodified PWS (Fig. 5b). The de-lignified material had the physical appearance of mechanical pulps, and readily absorbed all free water at the lowest solids concentrations, as seen by a simple visual examination. Similarly, there was no free water T_2_ peak present in the sample even at the lowest solids concentration. There was an immediate decrease in T_2_ times of the three major peaks already from the lowest solids concentration, and this decrease continued evenly until 30% DM, with the least constrained peak disappearing above 21% DM, most likely merging with the next peak. This corresponded well to the decrease in hydrolysis yields already from low-solids concentrations. At 30% DM, there were two major peaks at 9 and 24 ms, with the majority of water present in the less constrained of the two peaks, 31% and 68%, respectively, again indicating that the relative amount of water in the two major peaks relates to hydrolysis yields at high DM.

The xylanase-treated PWS, having the highest hydrolysis yields at high-solids concentrations, had the most distinct T_2_ profile as compared to the untreated PWS (Fig. 5c). The free water peak was present; however, it quickly disappeared above 10% DM, similar to the milled material. Besides the free water peak, 3 major water peaks appeared with the presence of a new peak at shorter T_2_ relaxation times compared to the unmodified PWS. At 30% DM, the two major pools of water were present, with slightly longer T_2_ times then for the unmodified material, which were 19 and 45 ms, respectively. The ratio between the amount of water in the shorter and longer T_2_ time peak for the xylanase-treated PWS was reversed as compared to the untreated PWS (Additional file 1: Table S1). Thus, there was a larger proportion of more constrained water in the system for the xylanase-treated PWS than for the untreated system, again confirming that more constrained water was related to higher hydrolysis yields at high-solids conditions.

To relate the studied system to a standard model for cellulose, as well as previous work studying cellulose–water interactions [19], water constraint and diffusion measurements were made for Avicel^®^ (PH 101), a commonly used model substrate for crystalline cellulose. T_2_ measurements (Fig. 5d) showed a striking difference to the behavior of the pretreated biomass samples. The free water peak (4 s) was shown to disappear by 18% DM, and there was only one major peak present above 18% DM which had a constant T_2_ time of 100 ms with increasing solids concentrations. This T_2_ is much longer than for water which would normally be associated with cell wall water, and therefore might be assigned to interstitial water between Avicel particles (this particular Avicel sample had a nominal average particle size of 50 mm). The reduction in the T_2_ time of the major peak with increasing solids concentrations was not seen at all for the Avicel samples, suggesting that the rigid structure of the material leads to a constant T_2_ profile with only diminishing peak intensity. This suggests that the reduction in T_2_ times of the different peaks in the pretreated lignocellulosic samples with increasing solids concentrations may be related to a shrinking of pores in the biomass matrix. The term pores is used in this work to describe the inter-polymer and inter-fiber space in the biomass matrix occupied by water.

### Diffusion of water in modified materials

**Fig. 6:**
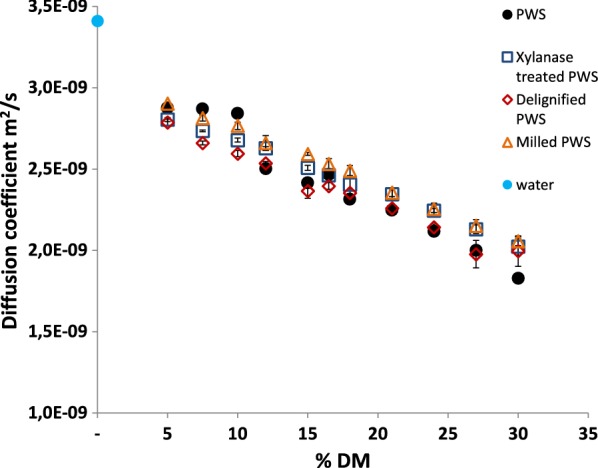
Diffusion coefficients measured for biomass–water slurries at different % DM for differently modified PWS material at 40 °C. value are the average of triplicate samples, and error bars represent ± standard deviation

Diffusion coefficients for water with increasing solids concentrations in the presence of the modified materials also decreased with increasing solids concentrations (Fig. 6). Diffusion coefficients were higher for the modified PWS materials than for the unmodified PWS at higher solids concentrations, however, no plateau effect was seen in the diffusion coefficients for the modified materials. This may have been due to the increased biomass–water interactions in general, and relatively less free water for the modified materials at low-solids concentrations. At lower solids concentrations, yields did not correlate to diffusion coefficients, and thus diffusion may not be a limiting factor for enzymatic hydrolysis at low-solids concentrations. At high-solids concentrations, the modified materials had higher diffusion rates, correlating to higher hydrolysis yields.

## Discussion

### Driving factors in the high-solids effect for enzyme preparations

Previous studies documenting the high-solids effect have shown an almost linear decrease with increasing solids concentration from even very low-solids concentrations using older cellulase preparations, which align well with results with the same enzyme preparations in this study [9, 10]. In this work, the high-solids effect, with the exception of de-lignified PWS, was not observed until above 15% DM when samples were hydrolyzed using the newer enzyme preparation (Ctec2). Previous work at lower solids concentrations with older cellulase preparations suggested increasing product inhibition to be the driving factor behind the high-solids effect [13]. This suggests that improved product inhibitor tolerance of the Ctec2 enzyme preparation leads to a later onset of the high-solids effect. If product inhibition of enzymes was the driving factor in the high-solids effect, reductions in yields would have been seen also at lower solids concentrations, as has been reported [13, 38]. Previous work has also suggested this to be the case [5], and the current study confirms this phenomenon. However, no effects of inhibitors generated during pretreatment can be inferred from the current study, as materials were washed prior to hydrolysis.

At lower solids concentrations, the PS material had a higher yield than the PWS material, while at the highest solids concentrations this relationship was reversed for hydrolysis reactions with Ctec2. Hydrolysis performance at low-solids concentrations is apparently not indicative of performance at higher solids concentrations, especially not above the threshold where free water disappears. That is: As long as free water is present, the biomass–water interactions during hydrolysis have no influence per se on sugar yields for a given substrate, i.e., performance is dominated by other factors such as for example lignin composition or which solutes are present in the liquid phase [14, 39, 40]. Above this threshold, the intrinsic solid biomass–water interactions become influential, and hydrolysis of substrates which allow water to stay or diffuse into the pores where hydrolysis is taking place is less negatively impacted. This is in agreement with our earlier finding that expansion of these pores due to diffusion of water, as a consequence of osmosis effects as sugar is introduced, can be a central factor for enzymatic degradation at high-solids concentrations [41].

For all samples, there was good agreement between the onset of the high-solids effect and the disappearance of free water peaks as seen using LFNMR. As well, decreases in internal diffusion coefficients for water corresponded well to decreases in hydrolysis yields for the unmodified pretreated materials. While the modified materials did not show this plateau effect or increased diffusion coefficients at lower solids concentrations, diffusion coefficients were higher for the modified materials at higher solids concentrations than for the unmodified PWS. This points to possibilities for improving mixing and mass transfer characteristics in process configurations [12], but also towards the possibility of modifying the biomass during the pretreatment to improve biomass–water interactions.

### Biomass characteristics and their relationship to water constraint profiles

LFNMR T_2_ profiles were different for the materials in this study, however they all responded similarly to increasing solids concentrations. The difference in the water constraint profiles and their behavior with increasing solids concentrations may be due to a number of factors and is open to different interpretations. Concentrating on the situation at high solids and assuming that each peak represents a specific range of pore sizes with a specific cell wall chemistry within the biomass matrix, one can speculate that the unmodified PWS has two main types of pore sizes/cell wall surface chemistries at these conditions, while PS has one main type of pore size/chemistry (Fig. 2). If pore surface chemistry is assumed to be constant with increasing solids concentrations, one can interpret the decrease in T_2_ times as a shrinking of the pores in the biomass matrix. This interpretation is in line with previous results for T_1_T_2_ NMR techniques showing decrease in both T_1_ and T_2_ times with increasing solids concentrations [41]. The PWS thus appears to possess two major pore sizes, which both shrink to a similar extent. However, this data could also be interpreted as two different pore cell wall chemistries, with one constraining water more than the other. The inability for T_2_ measurements to de-convolute chemical and physical effects of the biomass on water constraint makes it difficult to distinguish between these two hypotheses. If we look at the first interpretation in line with earlier T_1_T_2_ results, one would surmise that the more constrained water peak represents smaller pores, and thus relatively more exposed specific surface area is available for enzymatic action even at higher solids concentrations. Thus, as solids increase, the more constrained and conserved within the biomass matrix the water is, the more water exposed surface area is available for enzymatic hydrolysis, leading to improved yields relative to a system with larger pores and/or less constrained water. Also, as none of the major peaks disappeared from the system besides the free water peak, one might speculate that all the water is associated with the biomass matrix at high-solids. This behavior is similar to previous studies showing a reduction in peak time, and peak amplitude with decreasing water content on other types of pretreated biomass [36].

The post-pretreatment modifications to the PWS clearly changed the water constraint profiles. For the milled PWS material, the changes in the T_2_ profiles could be attributed to the increased available surface area caused by particle size reduction and higher levels of water constraint at low-solids concentrations (with more surface available to interact with the free water). This is supported by shorter T_2_ times and a quicker disappearance of the free water peak. As well, the size reduction and likely fibrillation of the PWS would lead to a reduction in the number of larger pores present in the material, and thus an increase in the relative amount of water present in the more constrained pool, as observed. This shows that physical changes of the biomass can have a significant effect on the water constraint of the system, and also the overall effect of high-solids concentration on hydrolysis yields. This again suggests that increased water constraint by the biomass relates to increased yields at high-solids conditions.

The de-lignified PWS sample, which had the largest change in chemistry from the untreated PWS, had a very different water constraint profile than the untreated PWS. As lignin is less hydrophilic than cellulose and restricts the available cellulose surface area, removing lignin from the biomass substrate exposes more cellulose, and lead to a greater degree of interaction between water and biomass. Furthermore, the cellulose micro-fibrils are less hindered by the lignin, and can swell to absorb the water present [42, 43]. This decrease in T_2_ times and increase in biomass–water interactions may also account for the increased hydrolysis yields recorded for the de-lignified PWS at low-solids concentrations, which is similarly related to accessible cellulose. However, as solids concentrations increased, a sharp decrease in the T_2_ times of the major peaks was seen, showing two peaks with roughly the same T_2_ times as previously identified in the unmodified PWS, with a slightly higher ratio of the amount of water in the shorter T_2_ peak to the longer T_2_ peak than for the unmodified material (Additional file 1: Table S1). Thus, even though a higher rate of decrease in hydrolysis yields and T_2_ times from lower solids concentrations was observed, the overall hydrolysis yield was improved as compared to the unmodified material, as has also been previously reported for lignin removing pretreatments [44]. The initial high yields at low-solids concentrations could also be attributed to less lignin blocking access to cellulose [45–47]. The sharp drop in T_2_ times with increasing solids concentrations may be related to less lignin being present to keep cellulose microfibrils from aggregating with one another. This would lead to a larger degree of pore collapse, and at high-solids concentrations result in a more compact material, which would explain also the ratio of water between the two constraint pools, with the more constrained pool being relatively less present due to the collapse of the material. Thus, from a process design point of view, it might be beneficial to preserve some of the more rigid materials (such as lignin) in pretreated biomass, to resist pore collapse and the concurrent decrease in sugar yields at higher solids concentrations.

The xylanase-treated PWS sample had the most unique water constraint profile, in comparison to the other modifications tested. At most solids concentrations, there were three major pools present (besides the free water pool), and in general the T_2_’s of the major peaks were shorter than the other modified samples. It could be hypothesized that removal of xylan led to more exposed cellulose, and this could account for the extra pool of constrained water, however it is not immediately apparent which pool of water could be related to this structural and compositional change. Regardless, while the chemical changes in the xylanase-treated material could not be directly related to specific changes in the T_2_ profiles, the important relationship between water constraint and yields is still apparent. With shorter T_2_ times at lower solids concentrations, there was a less sharp decrease in T_2_ times with increasing solids concentrations, which may relate to the less pronounced decrease in yields with increasing solids concentrations. This trend supports the interpretation that the sharper the decline in T_2_ times for the different pools, the greater the decrease in yields. T_2_ times at 30% DM for the xylanase-treated sample were comparable to the T_2_ times for the other samples, however, there was relatively more water in the more constrained peak. This again points to a higher portion of constrained water being important to higher cellulose conversion at high-solids concentrations and is therefore an indicator for comparing pretreated samples for their hydrolysis performance at high-solids concentrations.

The T_2_ water constraint profiles for Avicel, a highly crystalline and rigid cellulose, showed that the water pool associated with the biomass did not decrease significantly in T_2_ time with increasing solids concentration, but only decreased in relative intensity. This marked difference in behavior confirms that Avicel is a poor model material for cellulose as found in plant cell walls.

### Diffusion and the high-solids effect

The diffusion rate tracked generally with hydrolysis yields, confirming that mass transfer is one of the major underlying issues for high-solids hydrolysis reactions. Even small improvements in diffusion coefficients related to improvements in hydrolysis performance, suggesting that there might be possibilities for improving performance at high-solids concentrations by modifying mass transfer conditions. This could be achieved by recycling partially hydrolyzed material (with smaller particle sizes and better rheological properties), operating in a continuous fed batch mode, or by applying a surfactant which changed mass transfer and diffusion properties. However, these ideas remain to be tested, and therefore further integrated research is necessary.

### Different relative hydrolysis results for biomass at low and high-solids concentrations

Finally, it is worth again stating that comparing hydrolysis yields at low and high-solids concentrations led to different rankings in which was the most hydrolysable material. More simply put, the material which performed best at low-solids conditions was not the one that performed best at high-solids conditions. Thus, evaluation of the effect on biomass conversion yields of a given pretreatment needs to take place using a dry matter content relevant for the intended conversion conditions. It is also worth noting that this study was limited by the fact that all experiments were carried out on washed material, removing any effect of pretreatment-produced inhibitors or soluble polysaccharides on hydrolysis reactions. However, as the focus of this study was on changes to the insoluble fraction, it was deemed an acceptable simplification of the system.

Another limitation of this study is that it assumed a process configuration where pretreatment is carried out separately from enzymatic hydrolysis and fermentation, and where externally produced fungal cellulases are added to achieve hydrolysis of the biomass, followed by the fermentation organism. Other process configurations for biochemical conversion have been suggested, such as using cellulolytic enzyme producing fermentation organisms (so called consolidated bioprocessing). These types of processes may result in different effects of biomass water interactions at high solids concentrations on conversion efficiencies, and may have different mass transfer limitations due to the process for biomass deconstruction. Thus, the presented work is only applicable for processes which have enzymes added from external sources, and where pretreatment, hydrolysis, and fermentation occur separately. Thus, future work is needed to determine how biomass water interactions may play a role in consolidated bioprocesses.

## Conclusions

In this work, the high-solids effect was found to be primarily a function of biomass–water interactions, both through water constraint and diffusion in the biomass matrix. By modifying pretreated materials both physically and chemically, it was possible to improve hydrolysis yields at high-solids concentrations, and to reduce the intensity of the high-solids effect. The onset of the high-solids effect was found to correlate with the disappearance of free water from the reaction slurry, which was observable from LFNMR relaxometry measurements. At conditions below the solids concentration where free water disappeared, the Ctec2 enzyme preparations did not appear to be product inhibited, as yields remained constant. As solids concentration increased, those biomass samples which had more water in the highly constrained pool had better cellulose hydrolysis yields at high-solids concentrations. As well, the relative ranking of biomasses by their digestibility with enzymes changed from low to high-solids conditions, driven primarily by differences in biomass–water interactions. Pretreated materials should be compared at high-solids hydrolysis conditions if useful knowledge is to be gained about how these materials will function in an industrial high-solids setting. This work shows that understanding biomass–water interactions, and how they affect enzymatic processes for biomass deconstruction, is key to developing effective and efficient biomass conversion processes at high-solids concentrations.

## Methods

### Feedstocks and pretreatment

Wheat straw (*Triticum aestivum* L.) was steam pretreated in a continuous horizontal reactor (mini IBUS) at 195 °C for 18 min (PWS). The material was stored frozen, and thawed before use. Norway Spruce (*Picea abies* L.) was pre-impregnated with SO_2_ gas (2.5% wt./wt.) and pretreated in a 10L steam gun for 5 min at 210 °C as has been reported previously [37]. All materials were washed thoroughly before use in this study. Chemical composition (Table 1) was measured using standard NREL methods [48].

The Pretreated Wheat Straw material (PWS) was further modified for selected experiments investigating impacts of specific factors on the high-solids effect. The milled PWS material was prepared by taking the washed and wet PWS and grinding it in a standard basket type coffee grinder for two concurrent sessions of 10 s, for 20 s of total grinding time. De-lignified PWS was prepared using a sodium chlorite and acetic acid bleaching method previously reported [49]. 146 g of wet PWS (55 g dry basis) was added to an Erlenmeyer flask, to which 1668 g H_2_O was added, followed by 33 g of sodium chlorite, and then 33 ml of acetic acid. The mixture was allowed to react for 2 h with stirring at 350 rpm, after which time subsequent similar does of sodium chlorite and acetic acid were added, and the mixture was allowed to react for another 2 h. The mixture was then separated in a glass frit filtered vacuum flask and washed thoroughly before use. PWS was treated with Multifect Xylanase (Novozymes A/S) at 10% total solids and an enzyme loading of 100 mg/g cellulose in 50 mM citrate buffer at pH 5.0 for 48 h at 50 °C to remove a portion of the hemicellulose. The hydrolysis resulted in approximately 25% of the hemicellulose being removed, along with 8% of total glucan. The material was then deactivated by boiling at 100 °C for 20 min and washed thoroughly to remove residual enzyme protein from the sample. Composition for these materials is given in Table 1, not including the milled material, which was assumed to have the same composition as the PWS.

### Enzymatic hydrolysis

Enzymatic hydrolysis was carried out on the biomass samples using (unless otherwise noted) the Ctec2 cellulase preparation (Novozymes A/S) supplemented with a catalase as suggested previously [50]. Enzyme loadings were 15 mg enzyme preparation protein per gram cellulose, and 0.11 mg enzyme protein per gram cellulose for Ctec2 and catalase, respectively. This enzyme loading was used specifically such that there was a reasonable amount of cellulose hydrolysis, but that there would not be excess enzyme activity present such that positive changes to the enzyme performance could still be observed. As a comparison to the Ctec2 preparation, limited enzymatic hydrolysis experiments (data in Fig. 1) were carried out using a mixture of Celluclast 1.5L (a cellulase mixture, Novozymes A/S) and Novozyme188 (a β-glucosidase, Novozymes A/S) at a volumetric ratio of 5:1, and with a total protein loading of 45 mg enzyme protein per gram cellulose. While this enzyme loading was much higher than for the Ctec2 enzyme preparation, it was used to achieve comparable hydrolysis yields at low DM concentrations to the Ctec2 hydrolysis experiments, such that decreases in yield with increasing DM would be comparable. All hydrolysis reactions were carried out in triplicate in 20 ml plastic tubes in a temperature controlled tumbling reactor (internal diameter of 30 cm) designed to mimic freefall mixing, with a rotation speed of 30 rpm. Temperature was controlled to 50 C, and 50 mM (final concentration) citrate buffer at pH 5.1 was used to buffer the mixtures. Total solids concentration was varied between 5% and 30% DM using washed materials, with a total mass of 5 g for the reaction mixture. Hydrolysis reactions were carried out for 72 h, after which time samples were boiled for 20 min to deactivate the enzymes, and the slurry was diluted for glucose analysis. Glucose concentration was measured by HPLC using a Phenomenex Resex ROA column at 80 °C with 5 mM H_2_SO_4_ as eluent at a flow rate of 0.6 mL/min, with an RI detector. Cellulose conversion yields (into both glucose and cellobiose) were calculated based on total glucan content before hydrolysis, but after modification in the case of the de-lignified and xylanase-treated PWS samples, as given in Table 1, and samples were diluted by mass prior to HPLC analysis according to [51] to remove calculation errors for yield determinations at high-solids contents.

### NMR measurements

NMR measurements were made using a Bruker mq20 Minispec NMR with a fixed magnet (0.47T equal to 20 MHz), at 40 °C. T_2_ relaxation time measurements were made using a CPMG pulse sequence [28], with a pulse separation of between 0.05 and 0.25 ms (depending on the  % DM of the sample, with samples with lower % DM requiring longer pulse separations to allow for adequate signal decay time), and with between 12,000 and 32,000 echoes (again, depending on total signal decay time). Recycle delay was set to 10 s, and 32 scans were made for each measurement. Samples were prepared in triplicate, with varying DM contents between 5 and 30% DM. T_2_ Decay curves were analyzed using a non-negative least squares (NNLS) algorithm in a PROSPA software package (version 3.1) to extract population vs time curves for each of the samples, showing the relative amount of the different water populations with different T_2_ relaxation times [52].

Diffusion measurements of water were made using the same NMR with the gradient unit attached, at the standard temperature of 40 °C. Triplicate samples were prepared between 5 and 30% DM for each of the pretreated materials and equilibrated to 40 °C before measurement. The amplitudes of the spin-echoes of the samples were measured with and without pulsed field gradients applied between the two pulses (90° and 180°) at 5 different signal amplitudes between 25 and 85% relative field strength, with a gradient pulse length (δ) of 0.5 ms, the time between gradient pulses (Δ) as 7.5 ms. The self-diffusion coefficient (D) could then be measured according to the classic method suggested by Stejskal and Tanner [53], by plotting left and right sides of Eq. 1,

1 $$\ln \left( {\frac{{A_{G\left( t \right)} }}{{A_{G\left( 0 \right)} }}} \right) = \gamma^{2} \cdot D \cdot \delta^{2} \cdot \left( {\Delta - \frac{1}{3}\delta } \right) \cdot G^{2}$$

where A_G(t)_ is the amplitude of the signal at the echo with the gradient applied, A_G(0)_ is the signal amplitude without the gradient, G is the gradient field strength, and γ is the gyromagnetic ratio of the hydrogen nucleus (42.577 MHz per Tesla), and deriving the Diffusion coefficient D from the slope of the plot.

## Additional file

**Additional file 1: Table S1.** LF-NMR relaxometry data for pretreated materials, modified materials, and Avicel.

## Abbreviations

DM: dry matter concentration (wt dry matter/wt whole slurry) %
LFNMR: low field nuclear magnetic resonance
PWS: pretreated wheat straw
PW: pretreated spruce
C/N: Celluclast1.5L/Novozym188 enzyme mixture
NNLS: non-negative least squares
CPMG: Carr-Purcell-Meiboom-Gill pulse sequence
HPLC: high pressure liquid chromatography
