# Supplementary material for: Enzymatic hydrolysis is limited by biomass–water interactions at high-solids: improved performance through substrate modifications
Source: Biotechnol Biofuels. 2019 Jan 4;12:3. doi: 10.1186/s13068-018-1339-x (PMC6318902; doi:10.1186/s13068-018-1339-x)
Supplement: Supplementary file 1 — Additional file 1: Table S1. LF-NMR relaxometry data for pretreated materials, modified materials, and Avicel. [file 13068_2018_1339_MOESM1_ESM.pdf]

Additional file 1

Table S1: LF-NMR relaxometry data for pretreated materials, modified materials, and Avicel.

			Peak 1		Peak 2		Peak 3		Peak 4		Peak 5		Peak 6		Peak 7	
Substrate	DM %	mono-component T_2 time, ms	T_2 time, ms	% water in peak	T_2 time, ms	% water in peak	T_2 time, ms	% water in peak	T_2 time, ms	% water in peak	T_2 time, ms	% water in peak	T_2 time, ms	% water in peak	T_2 time, ms	% water in peak
PS	5	1,350			10	2%	48	8%	353	17%	1550	74%				
	10	731			7	1%	41	17%	233	24%	965	57%				
	12	624			14	1%	44	11%	232	22%	767	66%				
	15	445	5.1	0.3%	-		33	8%	179	23%	533	69%				
	16.5	307	2.5	0.3%	-		30	10%	160	32%	411	58%				
	18	62	5.3	1.0%	19	10%	55	76%	166	13%						
	21	55	3.3	0.4%	14	6%	46	76%	106	17%						
	24	40	2.2	0.9%	15	17%	40	71%	52	11%						
	27	34	4.5	2.5%	12	8%	30	90%	76	7%						
	30	27	5.0	1.4%	11	9%	26	93%	18	7%						
PWS	5	1,043	8	1.3%	-		38	4%	-		198	18%	1,160	77%		
	7.5	1,082	4	1%	20	3.2%	-		74	8%	315	33%	1,512	54%		
	10	986	4	0.9%	13	2.9%	35	7%	60	8%	231	34%	1,052	57%		
	12	118	4	1.5%	20	12%	-		79	55%	217	31%	-			
	15	81	3	1.2%	13	7.2%	-		52	54%	131	37%	-			
	16.5	91	4	0.9%	16	12.7%	-		60	55%	171	31%	-			
	18	63	5	3.8%	12	7.0%	-		40	45%	89	44%				
	21	48	7	5.9%	-		-		28	47%	69	47%	-			
	24	38	4	3.1%	-		-		19	37%	49	60%	-			
	27	31	3	2.4%	-		-		14	31%	39	66%	-			
	30	23	2	1.9%	-		-		11	28%	27	71%	-			

**PWS milled**	**5**	**833**	**-**	***0.0%***	**3**	***0.4%***	**10**	***3%***	**32**	***7%***	**212**	***21%***	**974**	***69%***		
	**7.5**	**491**	**0.8**	***0.7%***	**3**	***0.6%***	**13**	***7%***	**39**	***10%***	**180**	***29%***	**677**	***53%***		
	**10**	**264**	**0.5**	***0.9%***	**8**	***4.2%***	**-**		**26**	***15%***	**136**	***37%***	**405**	***43%***		
	**12**	**147**	**0.4**	***0.5%***	**3**	***1.2%***	**-**		**17**	***16%***	**69**	***30%***	**206**	***52%***		
	**15**	**107**	**0.4**	***1.0%***	**3**	***0.7%***	**-**		**15**	***16%***	**50**	***34%***	**159**	***49%***		
	**16.5**	**85**	**0.2**	***1.0%***	**1**	***0.9%***	**-**		**11**	***12%***	**36**	***35%***	**122**	***51%***		
	**18**	**76**	**0.1**	***0.9%***	**1**	***0.7%***	**-**		**10**	***10%***	**33**	***38%***	**110**	***50%***		
	**21**	**50**	**0.7**	***0.9%***	**6**	***0.9%***	**-**		**6**	***5%***	**22**	***43%***	**72**	***50%***		
	**24**	**38**	**0.4**	***1.1%***	**3**		**-**		**3**	***3%***	**17**	***44%***	**52**	***52%***		
	**27**	**30**	**0.3**	***1.3%***	**2**		**-**		**2**	***2%***	**13**	***42%***	**40**	***55%***		
	**30**	**22**	**0.5**	***2.2%***	**1**		**-**		**1**	***2%***	**10**	***35%***	**27**	***63%***		
**PWS de-lignified**	**5**	**382**	**1.07**	***0.5%***	**7**	***0.7%***	**30**	***3%***	**102**	***8%***	**321**	***73%***	**865**	***16%***		
	**7.5**	**206**	**0.35**	***0.2%***	**5**	***0.9%***	**26**	***5%***	**90**	***29%***	**219**	***54%***	**524**	***18%***		
	**10**	**186**	**0.56**	***0.5%***	**5**	***0.7%***	**20**	***4%***	**66**	***21%***	**167**	***57%***	**446**	***18%***		
	**12.0**	**145**	**0.88**	***0.2%***	**2**	***0.4%***	**11**	***2%***	**39**	***16%***	**112**	***55%***	**302**	***27%***		
	**15**	**98**	**0.16**	***1.2%***	**3**	***1.0%***	**8**	***1%***	**25**	***14%***	**69**	***55%***	**188**	***28%***		
	**16.5**	**95**	**-**		**3**	***1.1%***	**15**	***7%***	**28**	***23%***	**79**	***50%***	**226**	***24%***		
	**18**	**86**	**0.29**	***0.7%***	**2**	***0.9%***	**-**		**12**	***5%***	**42**	***47%***	**112**	***40%***		
	**21**	**61**	**0.49**	***1.5%***	**3**	***1.1%***	**-**		**16**	***15%***	**45**	***51%***	**124**	***31%***		
	**24**	**41**	**0.24**	***0.5%***	**3**	***1.8%***	**-**		**-**		**20**	***36%***	**52**	***62%***		
	**27**	**27**	**0.59**	***1.4%***	**-**		**-**		**-**		**11**	***19%***	**30**	***80%***		
	**30**	**20**	**0.63**	***1.3%***	**1**	***1.8%***	**-**		**-**		**9**	***31%***	**24**	***67%***		
**PWS xylanase**	**5**	**2384**	**-**		**6**	***1.4%***	**25**	***5%***	**69**	***9%***	**171**	***25%***	**424**	***13%***	**3,262**	**47%**
	**7.5**	**805**	**-**		**6**	***1.7%***	**28**	***7%***	**47**	***10%***	**138**	***48%***	**354**	***18%***	**2,833**	**21%**
	**10**	**200**	**-**		**4**	***1.1%***	**14**	***3%***	**42**	***13%***	**116**	***49%***	**260**	***26%***	**1,754**	**8%**
	**12**	**122**	**0.19**	**1%**	**5**	***1.5%***	**16**	***4%***	**41**	***17%***	**109**	***60%***	**262**	***16%***	**-**	
	**15**	**94**	**0.61**	**1%**	**3**	***1.1%***	**-**		**16**	***9%***	**56**	***46%***	**145**	***44%***	**-**	
	**16.5**	**80**	**0.56**	**1%**	**5**	***1.6%***	**7**	***2%***	**27**	***20%***	**74**	***62%***	**208**	***14%***	**-**	
	**18**	**67**	**0.76**	**1%**	**4**	***0.9%***	**7**	***1%***	**18**	***12%***	**52**	***59%***	**123**	***27%***	**-**	
	**21**	**60**	**0.36**	**1%**	**3**	***0.8%***	**5**	***2%***	**20**	***20%***	**54**	***62%***	**142**	***15%***	**-**	**-**
	**24**	**48**	**0.49**	**1%**	**5**	***2.2%***	**-**		**18**	***24%***	**44**	***56%***	**141**	***17%***	**-**	**-**
	**27**	**37**	**0.36**	**1%**	**2**	***0.9***	**3**	***2%***	**13**	***15***	**33**	***69***	**89**	***13***	**-**	**-**

						%				%		%		%		
	30	31	0.20	1%	2	2.1%	6	6%	-		19	59%	51	36%	-	-
Avicel	5	3,918	6	1%	17	2%	-		95	13%	354	1%	4,060	83%		
	7.5	3,719	6	2%	22	2%	-		98	21%	343	2%	3,952	74%		
	10	3,539	4	1%	16	3%	38	3%	109	31%	282	4%	3,951	60%		
	12	3,395	6	3%	28	5%	-		104	35%	286	4%	3,898	53%		
	15	3,110	6	3%	27	7%	-		101	43%	282	6%	3,862	41%		
	16.5	2,752	3	1%	12	6%	42	7%	107	47%	316	7%	3,757	32%		
	18	1,971	10	4%	34	7%	-		100	49%	230	15%	3,576	23%		
	21	1,682	3	2%	13	7%	25	4%	76	51%	179	19%	3,536	19%		
	24	NR	NR	NR	NR	NR	NR	NR	NR	NR	NR	NR	NR	NR		
	27	83	5	2%	19	8%	-	15%	55	38%	117	44%	893	3%		
	30	63	6	6%	13	10%	-	22%	67	75%		1%		0%		

Legend: T_2 times of major peaks and relative amount of water in each peak for pretreated and modified materials measured at different % DM. Calculated from the areas of each peak relative to total under peak area, after NNLS analysis of the T_2 decay curves. T_2 times are reported as the T_2 time at maximum peak height. Data represents the average of triplicate samples. Peak number is arbitrary, and defined as the peak with the fastest T_2 relaxation time being peak 1, and increasing in peak number from there. Peak numbers between different substrates are not comparable, as some substrates and samples have more or fewer peaks. This data is represented in Figures 2 and 5 in the manuscript. NR stands for not recorded.
